# Multi-Omics Analysis Provides Insights into a Mosaic-Leaf Phenotype of Astaxanthin-Producing Tobacco

**DOI:** 10.3390/plants14060965

**Published:** 2025-03-19

**Authors:** Jialin Wang, Zaifeng Du, Xiaoyang Lin, Peng Li, Shihao Sun, Changqing Yang, Yong Chen, Zhongfeng Zhang, Xue Yin, Ning Fang

**Affiliations:** 1 Key Laboratory of Synthetic Biology of Ministry of Agriculture and Rural Affairs, Tobacco Research Institute, Chinese Academy of Agricultural Sciences, Qingdao 266101, China; 2 No. 2 Department of Applied Research, Beijing Life Science Academy (BLSA), Beijing 102209, China

**Keywords:** astaxanthin, mosaic-like spots, chlorophyll metabolism, metabolomics, proteomics, small RNA transcriptomics

## Abstract

In metabolically engineered plants, the target products are usually uniformly distributed in the whole plant or specific tissues. When engineering tobacco to produce astaxanthin, a ketocarotenoid with strong antioxidant activity and multiple bioactivities, a scattered distribution of astaxanthin-producing regions was observed in a small portion of astaxanthin-producing tobacco plants, which caused mosaic-like red and green spots on the leaves (ASTA-mosaic). A physiological assay showed that the non-astaxanthin green region (Mosaic_G) had relatively higher chlorophyll content and better chloroplast structure than the astaxanthin-producing red region (Mosaic_R). Then, metabolomics, proteomics, and small RNA transcriptomics were employed to analyze the uneven distribution of astaxanthin-producing regions in tobacco leaves. The results of metabolomics and proteomics revealed a decrease in carotenoid metabolism, chlorophyll biosynthesis, and chlorophyll degradation in the Mosaic_G region. Pheophorbide a, an intermediate of chlorophyll degradation, was found to be significantly reduced in the Mosaic_G region, which was accompanied by the attenuation of chlorophyllase and pheophytinase, which catalyze the formation of pheophorbide a in chlorophyll degradation. Reductions in photosynthetic antenna proteins and photosystem-associated proteins were observed in the Mosaic_R region, consistent with the better chloroplast structure of the Mosaic_G region. Small RNA transcriptomics showed that several small RNAs could target chlorophyll-degradative genes, but they were more effective in targeting the astaxanthin biosynthetic genes. This finding was supported by the fact that the Mosaic_G region can remain green up to the senescence of tobacco leaves. This work provides insights into the mechanism of the uneven distribution of astaxanthin-producing regions in tobacco leaves and may contribute to the specialized utilization of tobacco plants for metabolic engineering.

## 1. Introduction

Astaxanthin is a red ketocarotenoid with extraordinary antioxidant activity and excellent coloring ability [1]. The antioxidant property of astaxanthin exhibits therapeutic significance in the treatment of cancer, cardiovascular disease, and diabetes [2,3,4]. The extensive application of natural astaxanthin in the food, nutritional supplements, cosmetics, and aquaculture industries contributes to its immense commercial value [5]. However, limited industrial resources and the high extraction costs of natural astaxanthin pose major obstacles to meeting market demand [6]. The elucidation of the biosynthetic pathway of astaxanthin and the emergence of metabolic engineering provide the potential to overcome these obstacles [7]. Natural astaxanthin can be biosynthesized de novo in some bacteria, algae, yeast, and several plant species [3,6]. Notably, several species within the *Adonis* genus are the only terrestrial plants capable of synthesizing astaxanthin de novo through a unique three-step reaction catalyzed by carotenoid β-ring-4-dehydrogenase (CBFD) and 4-hydroxy-β-ring-4-dehydrogenase (HBFD), which are distinct from the biosynthetic pathways in bacteria, algae, and yeast [8,9]. The biosynthesis pathway from β-carotene to astaxanthin in *Adonis aestivalis* is initiated with the addition of a hydroxyl group at the No. 4 carbon of the β-ring catalyzed by CBFD, continues with the dehydrogenation of this hydroxyl group to a carbonyl group catalyzed by HBFD, and terminates with the addition of a hydroxyl group at the No. 3 carbon of the same β-ring catalyzed by CBFD [8,9].

Plants, serving as excellent metabolic engineering platforms, have been employed for the production of astaxanthin in various species, including *Arabidopsis*, tobacco, tomato, corn, and potato, etc. [10,11,12,13,14,15]. These plant platforms often exhibit apparent reddening in specific tissues or entire plants due to the accumulation of astaxanthin [10,11,12,13,14,15,16,17,18]. The production of astaxanthin, a colored product of carotenoid biosynthesis, typically leads to uniform red distribution in tobacco plants, particularly in tobacco leaves [16,17,18]. However, some studies have discovered an uneven red distribution in astaxanthin-producing tobacco leaves [14,19]. Mortimer et al. [14] observed that the color at the leaf edges from one astaxanthin-producing tobacco line showed the most intense red coloration and gradually faded into green towards the leaf veins. Lu et al. [19] found that the leaves of heteroplasmic astaxanthin-producing tobacco exhibited green and red sectors rather than the uniform red observed in homoplasmic astaxanthin-producing tobacco plants. An uneven scattered color distribution characterized by mosaic-like red and green spots was also observed on the leaves of the astaxanthin-producing tobacco plants obtained in our previous work [20], which was a novel pattern of uneven distribution of astaxanthin-producing regions. Nevertheless, the mechanism of these uneven distributions of target products in astaxanthin-producing plants is still unclear. In addition, the production of astaxanthin caused a reduction in the chlorophyll content and an alteration of chloroplast structure [14,16,19,21], which is closely related to photosynthetic growth of plants [21]. The decreased chlorophyll content in astaxanthin-producing plants may be associated with perturbation in chlorophyll metabolism induced by astaxanthin biosynthesis [22]. Specifically, β-carotene, serving as the direct precursor of astaxanthin, originates from geranylgeranyl pyrophosphate (GGPP), which is also a precursor for chlorophyll [23,24]. The biosynthesis of astaxanthin may utilize the substances required for chlorophyll synthesis [16], and astaxanthin is mainly localized in the thylakoid membrane of astaxanthin-producing plants, influencing the function and stability of photosystems and ultimately leading to alterations in chloroplast structure, particularly in the stacking of grana membranes [21]. Whether this uneven distribution of astaxanthin-producing regions is correlated with the mechanism of mitigating growth stress through regulating chlorophyll metabolism and chloroplast structure remains unknown. Overall, the uneven distribution of astaxanthin-producing regions in tobacco leaves directly affects the efficiency of astaxanthin production in metabolic engineering plants. Therefore, exploring the reason behind this phenomenon is of great significance for optimizing metabolic engineering strategies for the biosynthesis of astaxanthin. Furthermore, this uneven distribution of astaxanthin-producing regions may inspire the specialized utilization of tobacco plants for metabolic engineering in the future.

In this study, we analyzed the astaxanthin-producing red region (Mosaic_R) and non-astaxanthin green region (Mosaic_G) on the leaves of astaxanthin-producing tobacco obtained from previous work [20] through a physiological assay and multi-omics analysis. The physiological assay revealed that the Mosaic_G region exhibited higher chlorophyll content and better chloroplast structure compared to the Mosaic_R region. Further metabolomics and proteomics showed the attenuation of the carotenoid metabolism, chlorophyll synthesis, and chlorophyll degradation pathways, as well as an increase in photosynthetic antenna proteins and photosystem-associated proteins in the Mosaic_G region. Small RNA transcriptomics showed that small RNAs appeared to exert an effect on the attenuation of the chlorophyll-degradative genes, which seemed to be more effective on astaxanthin biosynthetic genes in the Mosaic_G region. Notably, the Mosaic_G region remained green on senescent tobacco leaves, and the uneven distribution of astaxanthin-producing regions was capable of stable inheritance. This work provides insights into the mechanisms underlying the uneven distribution induced by astaxanthin.

## 2. Results

### 2.1. Characterization of Astaxanthin-Producing Tobacco Plants with a Scattered Distribution of Astaxanthin-Producing Regions (ASTA-Mosaic)

**Figure 1:**
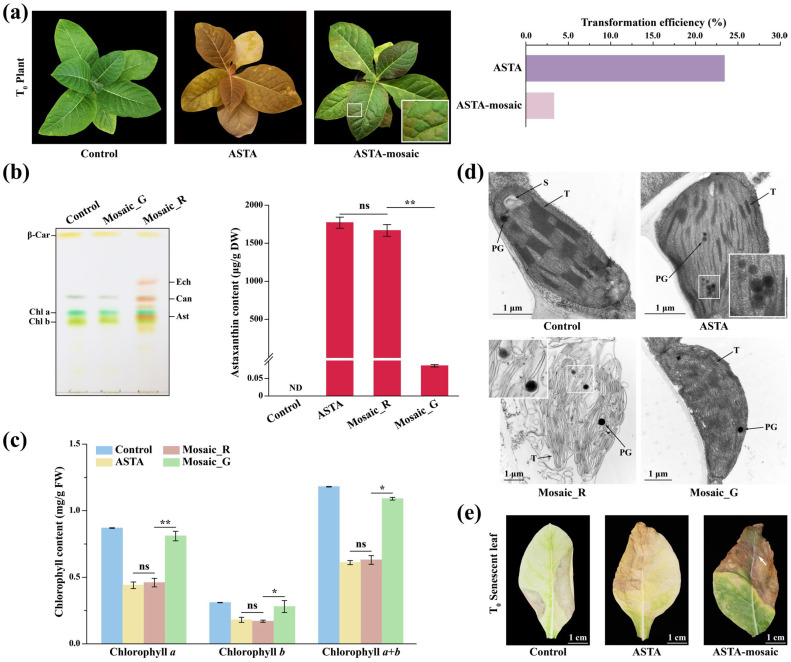
A scattered distribution of astaxanthin-producing regions in astaxanthin-producing tobacco leaves. (**a**) Phenotype of control, red astaxanthin-producing plant, and ASTA-mosaic plant (left) and transformation efficiency of red astaxanthin-producing plants and ASTA-mosaic plants in the process of transgenic cultivation (right). (**b**) TLC analysis of pigments in the Mosaic_R and Mosaic_G regions (left) and UPLC analysis of astaxanthin content in control plants, red astaxanthin-producing plants, Mosaic_R regions and Mosaic_G regions. β-Car, β-carotene; Chl a, chlorophyll a; Chl b, chlorophyll b; Ech, echinenone; Can, canthaxanthin; Ast, astaxanthin. ND, not detected. Asterisks indicate significant difference according to Student’s *t*-test (ns, *p* > 0.05; **, *p* < 0.01). (**c**) Chlorophyll content in control plants, red astaxanthin-producing plants, Mosaic_R regions and Mosaic_G regions. Asterisks indicate significant difference according to Student’s *t*-test (ns, *p* > 0.05; *, *p* < 0.05; **, *p* < 0.01). (**d**) Chloroplast ultrastructure of the Mosaic_R and Mosaic_G regions. S, starch; T, thylakoid; PG, plastoglobuli. (**e**) Senescent leaf of control, red astaxanthin-producing plant, and ASTA-mosaic plant. White arrows indicate Mosaic_G region. ASTA, red astaxanthin-producing plant.

In our previous work [20], a minority of astaxanthin-producing tobacco plants exhibited a scattered distribution of astaxanthin-producing regions, leading to mosaic-like red and green spots on the leaves (Figure 1a). A random Mosaic_G region gradually emerged on the leaves of ASTA-mosaic plants during tissue culture and expanded further as the plants matured, which extended even to the petal, calyx, and seed capsule (Figure 1a and Figure S1A,B). Approximately 3% of the transgenic plants displayed an uneven distribution of astaxanthin-producing regions, accounting for 14% of astaxanthin-producing tobacco plants (Figure 1a). Subsequent thin-layer chromatography (TLC) analysis confirmed the absence of astaxanthin in the Mosaic_G region (Figure 1b), and an ultra-performance liquid chromatography (UPLC) assay further revealed that the astaxanthin content in the Mosaic_G region significantly decreased, while the astaxanthin content in the Mosaic_R region was similar to that in red astaxanthin-producing tobacco plants (Figure 1b). Astaxanthin production has been demonstrated to cause chlorophyll reduction and chloroplast structure alteration [14,21]. Consequently, the absence of astaxanthin should result in the restoration of chlorophyll content and chloroplast structure. Measurements of chlorophyll content showed a significant increase in the Mosaic_G region compared to the Mosaic_R region, similar to the level in the control plants (Figure 1c), and transmission electron microscopy (TEM) was then used to verify the changes in the chloroplast ultrastructure of the Mosaic_R and Mosaic_G regions. In contrast to the control plants, the Mosaic_R region exhibited changes in chloroplast ultrastructure akin to those observed in red astaxanthin-producing tobacco plants, including a decrease in thylakoid abundance and thylakoid stacking and an increase in plastoglobuli (Figure 1d). Conversely, the Mosaic_G region showed an increase in thylakoid abundance and thylakoid stacking and a reduction in plastoglobuli (Figure 1d). Interestingly, part of the Mosaic_G region seemed to maintain green during leaf senescence (Figure 1e and Figure S1C), and this scattered distribution of astaxanthin-producing regions on the leaves of ASTA-mosaic plants could be stably inherited by the next generation (Figure S1D).

### 2.2. Untargeted Metabolomics Analysis of Mosaic_R and Mosaic_G Regions

**Figure 2:**
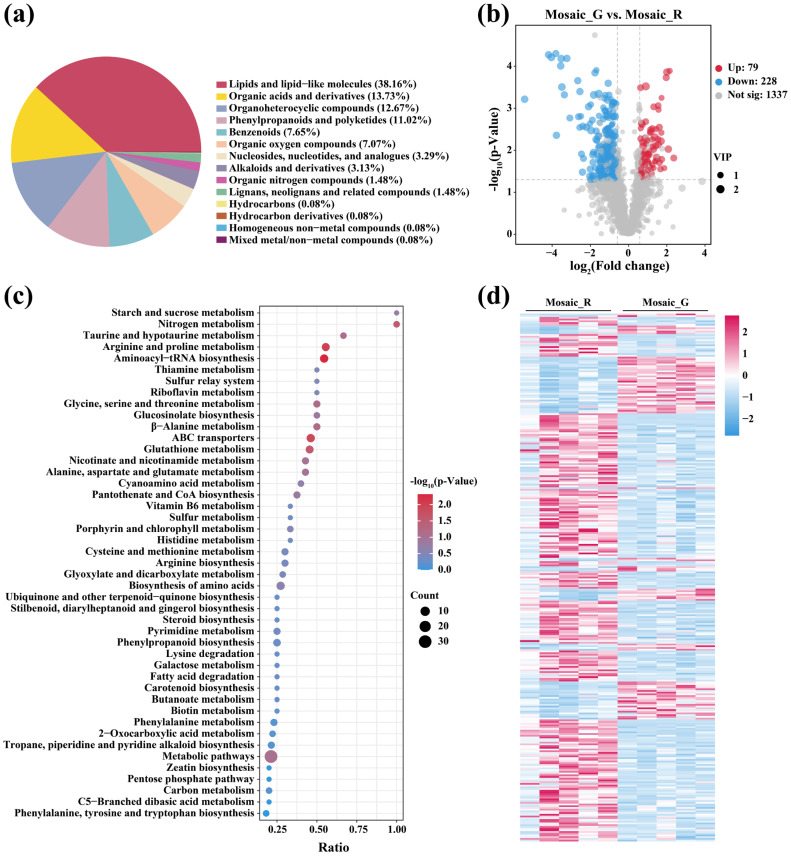
Untargeted metabolomics analysis of Mosaic_R and Mosaic_G regions. (**a**) Classification of identified metabolites between the Mosaic_R and Mosaic_G regions. (**b**–**d**) Volcano plot (**b**), KEGG pathway analysis (**c**), and heatmap (**d**) of the DCMs between the Mosaic_R and Mosaic_G regions. VIP, variable importance in projection.

Untargeted metabolomics analysis was performed to investigate the metabolic level differences between the Mosaic_R and Mosaic_G regions. A total of 1644 identified metabolites were classified into lipids and lipid-like molecules (38.16%), organic acids (13.73%), organoheterocyclic compounds (12.67%), phenylpropanoids and polyketides (11.02%), benzenoids (7.65%), organic oxygen compounds (7.07%), nucleosides, nucleotides, and analogues (3.29%), alkaloids (3.13%), organic nitrogen compounds (1.48%), lignans, neolignans and related compounds (1.48%), hydrocarbons (0.08%), hydrocarbon derivatives (0.08%), homogeneous non-metal compounds (0.08%), and mixed metal/non-metal compounds (0.08%) (Figure 2a and Table S1). Among the identified metabolites, a total of 307 differentially changed metabolites (DCMs) were detected, with 79 upregulated DCMs and 228 downregulated DCMs in the Mosaic_G region (Figure 2b and Table S2). Kyoto encyclopedia of genes and genomes (KEGG) pathway analysis showed that 307 DCMs were enriched into 45 pathways, 2 DCMs were enriched in the porphyrin and chlorophyll metabolism, which were L-glutamic acid and pheophorbide a, and 1 DCM was enriched in the carotenoid biosynthesis, which was astaxanthin (Figure 2c). Furthermore, the heatmap shows the changes in the DCMs between the Mosaic_R and Mosaic_G regions (Figure 2d). These findings indicate significant metabolic differences between the Mosaic_R and Mosaic_G regions.

### 2.3. Tandem Mass Tags (TMT)-Based Quantitative Proteomics Analysis of Mosaic_R and Mosaic_G Regions

**Figure 3:**
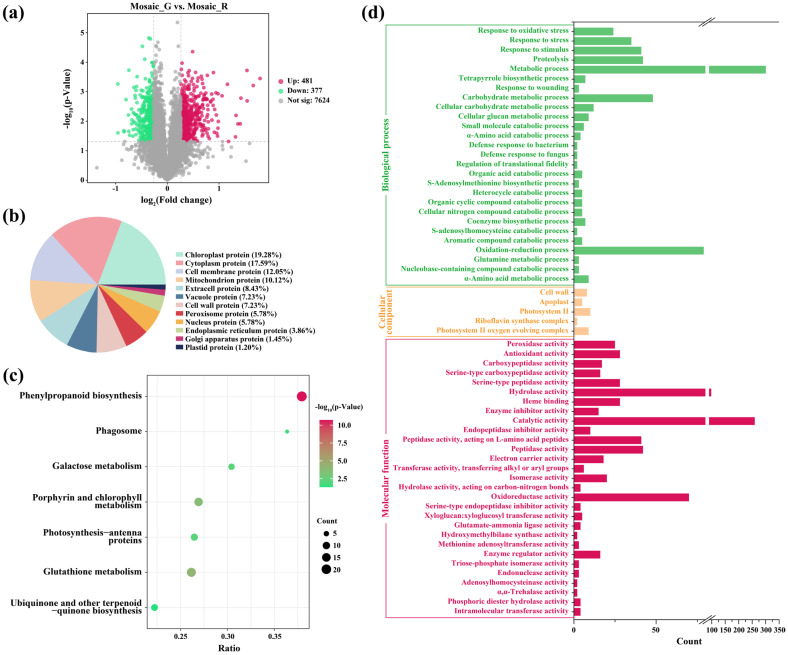
TMT-based quantitative proteomics analysis of the Mosaic_R and Mosaic_G regions. (**a**–**d**) Volcano plot (**a**), subcellular localizations (**b**), KEGG pathway analysis (**c**), and GO enrichment (**d**) of DAPs between the Mosaic_R and Mosaic_G regions.

TMT-based quantitative proteomics analysis was conducted to explore the differences of protein level between the Mosaic_R and Mosaic_G regions, in which a total of 858 differentially accumulated proteins (DAPs) were identified in the 8482 detectable proteins, including 481 upregulated DAPs and 377 downregulated DAPs (Figure 3a and Tables S3 and S4). The subcellular localizations of the DAPs were primarily concentrated in chloroplast proteins (19.28%) (Figure 3b). The KEGG pathway analysis of DAPs revealed that 1.6% of the DAPs were enriched in the porphyrin and chlorophyll metabolism and 1.0% of DAPs were enriched in the photosynthesis antenna protein (Figure 3c). Gene ontology (GO) enrichment analysis showed that the photosystem II and photosystem II oxygen evolving complex accounted for the largest proportion in the cellular components category (Figure 3d). These results imply that there are considerable fluctuations in the accumulation of proteins related to chlorophyll and chloroplasts, corresponding to the restoration of chlorophyll and chloroplast structure in the Mosaic_G region.

### 2.4. The Changes of Carotenoid Metabolism, Chlorophyll Metabolism, and Photosystem Structure in the Mosaic_R and Mosaic_G Regions

**Figure 4:**
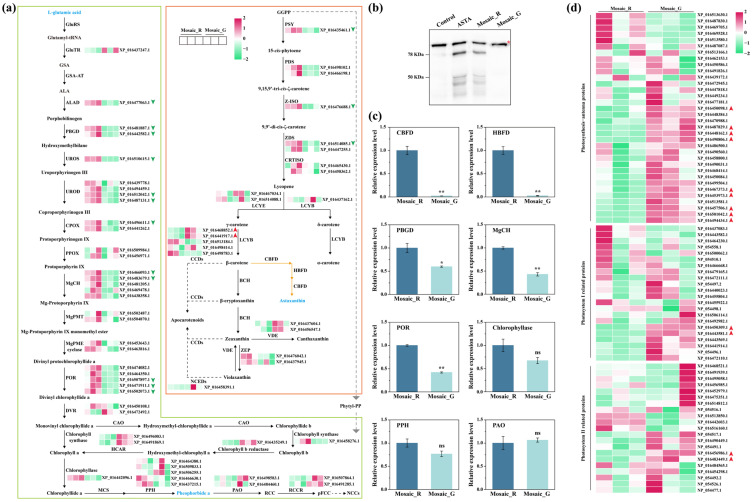
Changes in chlorophyll metabolism, carotenoid metabolism, and chloroplast structure between the Mosaic_R and Mosaic_G regions: (**a**) Summary of proteins and metabolites changes in the chlorophyll and carotenoid metabolism between the Mosaic_R and Mosaic_G regions. Blue font indicates downregulated DCMs. Red and green arrows indicate upregulated and downregulated DAPs, respectively. The green box and orange box indicate the chlorophyll metabolism pathway and carotenoid metabolism pathway, respectively. The orange arrow shows the biosynthetic pathway of astaxanthin. (**b**) Western blotting analysis of astaxanthin synthases in the Mosaic_R and Mosaic_G regions. Red asterisk indicates non-specific protein bands. (**c**) Gene relative expression levels of CBFD, HBFD, and proteins involved in chlorophyll metabolism. Asterisks indicate significant difference between Mosaic_R and Mosaic_G regions according to Student’s *t*-test (ns, *p* > 0.05; *, *p* < 0.05; **, *p* < 0.01). (**d**) Heatmap of proteins related to the photosystem structure between the Mosaic_R and Mosaic_G regions. Red arrows indicate upregulated DAPs. GSA, glutamate 1-semialdehyde; ALA, 5-aminolevulinic acid; RCC, red chlorophyll catabolite; pFCC, primary fluorescent chlorophyll catabolite; NCCs, nonfluorescent chlorophyll catabolites; GGPP, geranylgeranyl pyrophosphate; GluRS, glutamyl-tRNA synthetase; GluTR, glutamyl-tRNA reductase; GSA-AT, glutamate-1-semialdehyde aminotransferase; ALAD, aminolevulinic acid dehydratase; PBGD, porphobilinogen deaminase; UROS, uroporphyrinogen III synthase; UROD, uroporphyrinogen III decarboxylase; CPOX, coproporphyrinogen oxidase; PPOX, protoporphyrinogen oxidase; MgCH, magnesium chelatase; MgPMT, Mg-protoporphyrin IX methyltransferase; MgPME cyclase, Mg-protoporphyrin IX monomethyl ester cyclase; POR, protochlorophyllide reductase; DVR, 3,8-divinyl protochlorophyllide a 8-vinyl reductase; CAO, chlorophyllide a oxygenase; HCAR, 7-hydroxymethyl chlorophyll a reductase; MCS, metal-chelating substance; PPH, pheophytinase; PAO, pheophorbide a oxygenase; RCCR, red chlorophyll catabolite reductase; PSY, phytoene synthase; PDS, phytoene desaturase; Z-ISO, 15-cis-ζ-carotene isomerase; ZDS, ζ-carotene desaturase; CRTISO, carotenoid isomerase; LCYE, lycopene epsilon-cyclase; LCYB, lycopene β-cyclase; BCH, β-carotene hydroxylase; CCDs, carotenoid cleavage dioxygenases; VDE, violaxanthin de-epoxidase; ZEP, zeaxanthin epoxidase; NCEDs, 9-cis-epoxycarotenoid dioxygenases.

β-carotene originates from GGPP, which is the precursor for the synthesis of astaxanthin, as well as the phytol-tail of chlorophyll [23,24]. β-carotene is excessively consumed for astaxanthin synthesis in the astaxanthin-producing tobacco plants [19], potentially leading to an enhancement in β-carotene synthesis, even the entire carotenoid metabolism. The downregulation of most proteins involved in the carotenoid metabolism in the Mosaic_G region suggested that the biosynthesis of astaxanthin substantially reduced or even ceased (Figure 4a and Table S5). Further Western blotting analysis and transcription assay via the quantitative real-time PCR (qRT-PCR) testing of CBFD and HBFD confirmed that the decrease in astaxanthin biosynthesis was attributable to the absence of CBFD-HBFD fusion protein expression in the Mosaic_G region (Figure 4b,c). Simultaneously, the significant reduction in the accumulation of L-glutamic acid, a precursor of chlorophyll synthesis [25], coupled with the downregulation of protein expression levels in the chlorophyll synthesis at the Mosaic_G region, indicated attenuated chlorophyll synthesis compared to the Mosaic_R region (Figure 4a and Table S5). The protein abundance associated with chlorophyll degradation exhibited an unclear trend in the Mosaic_G region (Figure 4a and Table S5). Porphobilinogen deaminase (PBGD), magnesium chelatase (MgCH), and protochlorophyllide reductase (POR), enzymes of chlorophyll synthesis, were then selected for proteomics validation using qRT-PCR. The results showed that the expression levels of *PBGD*, *MgCH*, and *POR* significantly decreased, consistent with proteomics findings (Figure 4c). Pheophorbide a, an intermediate of chlorophyll degradation [25], notably decreased in the Mosaic_G region, potentially leading to the accumulation of upstream products in chlorophyll degradation or the attenuation of chlorophyll degradation, thereby exerting feedback inhibition on chlorophyll synthesis (Figure 4a and Table S5). Transcription assay and enzyme activity analysis were conducted to investigate the reduction in pheophorbide a, which was attributed to either the diminution of upstream chlorophyllase and pheophytinase (PPH) or the enhancement of downstream pheophorbide a oxygenase (PAO). The results revealed the attenuation of chlorophyllase and PPH in the Mosaic_G region, while there was no significant alteration in PAO (Figure 4c and Figure S2), which indicated that the decreased accumulation of pheophorbide a was attributable to the attenuation of chlorophyll degradation. Furthermore, the abundance of over 50% of the photosynthesis antenna proteins (chlorophyll *a*/*b*-binding proteins), which could maintain the structure of thylakoid membrane [26], as well as some proteins related to photosystem I and II, increased in the Mosaic_G region (Figure 4d and Table S5). These findings indicate a decrease in carotenoid metabolism and chlorophyll synthesis, as well as chlorophyll degradation, as evidenced by the reduced accumulation of pheophorbide a resulting from the attenuation of chlorophyllase and PPH within the Mosaic_G region. And the upregulation of photosynthetic antenna proteins and some proteins related to photosystems is consistent with the more optimal chloroplast structure in the Mosaic_G region.

### 2.5. Small RNA Transcriptomics Analysis of Mosaic_R and Mosaic_G Regions

**Figure 5:**
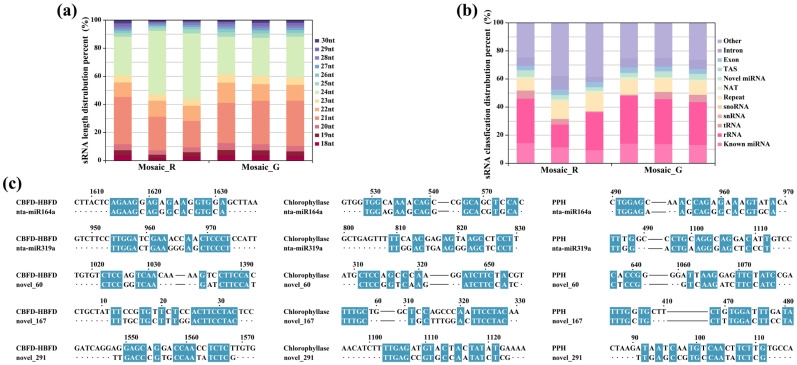
Small RNA transcriptomics analysis of Mosaic_R and Mosaic_G regions. (**a**,**b**) Length (**a**) and classification distribution (**b**) of small RNAs in Mosaic_R and Mosaic_G regions. NAT, natural antisense transcript; TAS, ta-siRNA. (**c**) Sequence alignment between DERs and genes of astaxanthin synthases (CBFD-HBFD), chlorophyllase, and PPH. Blue box indicates aligned sequence between DERs and indicated genes.

The findings of metabolomics and proteomics demonstrated a decrease in carotenoid metabolism, chlorophyll synthesis, and chlorophyll degradation within the Mosaic_G region. Small RNA transcriptomics analysis was employed to further investigate the causes underlying the scattered distribution of astaxanthin-producing regions. Six small RNA libraries were constructed from the Mosaic_R and Mosaic_G regions, with the length distribution of small RNAs in them mainly being concentrated at 21 nt and 24 nt (Figure 5a). The principal classifications of small RNAs in both the Mosaic_R and Mosaic_G regions were rRNA and other RNA (Figure 5b). Subsequently, differential analysis was then performed on all miRNAs, encompassing known miRNAs and novel miRNAs. Five differentially expressed miRNAs (DERs), which were nta-miR164a, nta-miR319a, novel_60, novel_167, and novel_291, were targeted to 875 genes (Table S6), which were enriched in 57 different pathways (Table S7). Afterwards, sequence alignment between the five DERs and the gene sequences of astaxanthin synthases (CBFD-HBFD), chlorophyllase, and PPH showed that all five DERs had high sequence identity with astaxanthin synthases, and novel_291 exhibited a higher sequence identity to chlorophyllase and PPH than the other four DERs (Figure 5c). These results imply that small RNAs could target the chlorophyll degradation genes, which is supported by the observation that the Mosaic_G region can remain green up to the senescence of tobacco leaves. However, small RNAs appear to be more effective in targeting the astaxanthin biosynthetic genes in the Mosaic_G region.

## 3. Discussion

Some metabolically engineered tobacco plants for astaxanthin biosynthesis exhibited uneven distribution of red and green colorations on the leaves, indicating unevenly distributed astaxanthin production [14,19]. This study revealed that the non-astaxanthin regions exhibited higher chlorophyll content and better chloroplast structure compared to the astaxanthin-producing regions. Moreover, decreased carotenoid metabolism and chlorophyll synthesis, as well as chlorophyll degradation supported by reduced pheophorbide a, were observed in the Mosaic_G region, whereas the abundance of photosynthetic antenna proteins and photosystem-associated proteins increased. Furthermore, several small RNAs could target the genes of chlorophyllase and PPH involved in chlorophyll degradation, which were more effective in targeting the astaxanthin biosynthetic genes. Interestingly, some Mosaic_G regions retained the green color on senescent leaves. This work provides insights into the mechanism underlying the uneven distribution of astaxanthin-producing regions in tobacco leaves.

Many astaxanthin-producing plants display growth retardation, accompanied by alterations in chlorophyll content and chloroplast structure, which are crucial components of photosynthetic growth in plants [12,19,27]. The significant changes in chlorophyll content and chloroplast structure observed in the Mosaic_R region, which are consistent with other studies [14,21], likely reflects chloroplast disorganization due to astaxanthin production. Concurrently, the decreased abundance of photosynthetic antenna proteins and photosystem-associated proteins in the Mosaic_R region also corroborates this finding. In contrast, the emergence of the Mosaic_G region, characterized by high chlorophyll content and optimal chloroplast structure, may represent an adaptive response in astaxanthin-producing tobacco plants to mitigate growth retardation, suggesting an effective strategy of increasing chlorophyll content or maintaining chloroplast structure in astaxanthin-producing plants to enhance plant growth.

The reduced accumulation of pheophorbide a, which is a degradation product of chlorophyllide a [25], correlates with diminished activities of chlorophyllase and PPH in the Mosaic_G region, indicating an attenuation of chlorophyll degradation. MCS, a smaller heat-stable substance participated in the non-enzymatic reaction of removing Mg from chlorophyllide a to form pheophorbide a [28], may also contribute to the attenuation of chlorophyll degradation, which requires more evidence to support in the future. Moreover, the attenuation of chlorophyll degradation can lead to the accumulation of one of its intermediates, most likely chlorophyllide a, and negatively regulate the chlorophyll synthesis, which also explains the attenuation of the chlorophyll synthesis observed in the Mosaic_G region.

Lu et al. [19] utilized plastid transformation to express bacterial-derived astaxanthin synthases in the tobacco chloroplast genome and observed an uneven distribution of astaxanthin-producing regions in the astaxanthin-producing tobacco plants, which resulted from the incomplete replacement of wild-type chloroplast copies by transformed chloroplast copies (referred to as heteroplasmic). Heteroplasmic transplastomic plants gradually achieve homoplasmy into either uniform red astaxanthin-producing tobacco plants or green wild-type tobacco plants during cultivation [29]. However, the ASTA-mosaic plants did not exhibited a transition from heteroplasmy to homoplasmy during cultivation. And the ASTA-mosaic plants were obtained by nuclear transformation method, because the astaxanthin synthases from *A. aestivalis* were predicted to contain chloroplast transit peptides. Considering the differences in the transformation method and the distribution pattern of astaxanthin-producing regions, the uneven distribution of astaxanthin-producing regions may originate from the co-suppression induced by small RNA [30,31]. The appearance of the Mosaic_G region could be a strategy to defend against foreign nucleic acid intrusion and preserve genomic structural integrity in astaxanthin-producing tobacco plants [32], and the simultaneous attenuation of astaxanthin biosynthesis and chlorophyll degradation in the Mosaic_G region may be mediated by the aforementioned small RNAs. The high sequence homology between these small RNAs and astaxanthin biosynthetic genes suggests a preferential suppression of astaxanthin biosynthesis. Furthermore, the phenomenon of the Mosaic_G region remaining green on senescent tobacco leaves may also be associated with the targeted inhibition of chlorophyll degradation by small RNAs.

Interestingly, some Mosaic_G regions were observed to stay green on senescent leaves. These stay-green mutants have been identified in many plant species and classified into two categories: functional and cosmetic mutants, determined by the retention or absence of photosynthetic activity in the green regions [33]. And the functional stay-green mutants have the potential to increase plant biomass, while the cosmetic stay-green mutants are likely to be defective in chlorophyll degradation [33]. Based on the results of metabolomics, proteomics, and small RNA transcriptomics, the inhibition of chlorophyll degradation in the Mosaic_G region indicates that the ASTA-mosaic tobacco plants might be cosmetic stay-green mutants, which are highly beneficial in elucidating the process of chlorophyll degradation in tobacco [33]. However, the hypothesis that ASTA-mosaic tobacco plants are functional stay-green mutants cannot be overlooked, which is more advantageous for tobacco, whose biomass primarily relies on leaves [34].

In summary, this study found a scattered distribution of astaxanthin-producing regions in astaxanthin-producing tobacco leaves, and the non-astaxanthin regions had higher chlorophyll content and better chloroplast structure compared to the astaxanthin-producing regions. This study also revealed a diminished carotenoid metabolism and chlorophyll synthesis, as well as chlorophyll degradation evidenced by the reduction in pheophorbide a in the Mosaic_G region through metabolomics and proteomics. Furthermore, increases in photosynthetic antenna proteins and photosystem-associated proteins were also observed in the Mosaic_G region, consistent with the better chloroplast structure of the Mosaic_G region. Small RNA transcriptomics further confirmed the inhibitory effect of small RNAs on astaxanthin biosynthetic genes and chlorophyll-degradative genes in the Mosaic_G region. Notably, some Mosaic_G regions in the senescent leaves remained green. Overall, this work offers insights into the potential mechanism of the uneven distribution of astaxanthin-producing regions in astaxanthin-producing plants, which may contribute to optimizing metabolic engineering strategies for the biosynthesis of astaxanthin and inspire the specialized utilization of tobacco plants for metabolic engineering.

## 4. Materials and Methods

### 4.1. Cultivation of Plant Material

The 5-week-old ASTA-mosaic plants were obtained from previous work [20]. All the plant materials were cultivated in an indoor greenhouse at 23 °C with a photoperiod of 14 h/10 h.

### 4.2. Statistical Analysis of Transformation Efficiency

The transformation efficiency was defined as the ratio of the number of positive transgenic plants to the total number of leaf discs.

### 4.3. Identification and Quantification of Related Pigment by TLC

A total of 0.2 g of fresh leaves were extracted with 1.5 mL acetone. The supernatant, after centrifugation, was the mixture of pigments and separated on a silica gel plate (Macklin, Shanghai, China) in the TLC development tank with mixed developing solvent (petroleum ether/isopropanol/water 100/10/0.1, *v*/*v*/*v*).

### 4.4. Measurement of Astaxanthin in Tobacco

Measurement of astaxanthin content from tobacco leaves was performed via UPLC assay. The Mosaic_R and Mosaic_G regions were carefully cut and collected from astaxanthin-producing tobacco leaves with a sharp blade. A total of 0.5 g of fresh leaf material was ground into a fine powder after freeze-drying and extracted with acetone. A total of 2 mL extract was dried under a nitrogen flow in the dark at room temperature and redissolved in 1 mL methanol with 10 μL trichloromethane. Redissolved solute was then subjected to an ACQUITY UPLC^TM^ system (Waters, Milford, MA, USA) for the UPLC assay after passing through a 0.22 μm filter. The column was a BEH C18 chromatographic column (Waters, Milford, MA, USA), and the column temperature was set at 35 °C. The gradient elution system is shown in Table S8. And astaxanthin standard reference came from Sigma-Aldrich (Darmstadt, Germany).

### 4.5. Measurement of Chlorophyll Content in Tobacco

The Mosaic_R and Mosaic_G regions were carefully cut and collected from fresh astaxanthin-producing tobacco leaves. A total of 0.1 g of fresh leaf samples were soaked in 5 mL 95% ethanol for 2 days in the dark. The absorbance of 200 µL chlorophyll extract was measured on a BioTek Synergy LX Multimode Reader (Agilent, Santa Clara, CA, USA) at 649 nm and 665 nm. The chlorophyll content was calculated by applying the following formulae.

chlorophyll *a* (mg/g) = (13.95 × A665 − 6.88 × A649) × (5/1000)/fresh weight (1)

chlorophyll *b* (mg/g) = (24.96 × A649 − 7.32 × A665) × (5/1000)/fresh weight (2)

chlorophyll *a* + *b* (mg/g) = (6.63 × A665 + 18.08 × A649) × (5/1000)/fresh weight (3)

### 4.6. Observation of Chloroplast Ultrastructure via TEM

The chloroplast ultrastructures of the fresh leaf samples were observed using TEM. Fresh leaf samples were cut into ~1 mm^2^ pieces and fixed in 2.5% glutaraldehyde. Then, the samples were washed with 0.1 M phosphate buffer (pH 7.2) and refixed in 2% osmium acid. The samples were subsequently subjected to gradual dehydration using a series of gradient ethanol and embedded in resin EPON 812. The embedded sample sections were prepared via Reichert–Jung ULTRACUT E ultramicrotome (Leica, Vienna, Austria) and counterstained with uranyl acetate, followed by lead citrate. The samples were observed and imaged by JEM1200 transmission electron microscope (JEOL, Tokyo, Japan).

### 4.7. Untargeted Metabolomics Analysis

Untargeted metabolomics analysis was conducted by Novogene company (Beijing, China). In short, fine tobacco powder was extracted with 80% methanol, and the supernatant of the extract was then diluted to 53% methanol for UPLC-MS/MS analysis via a Vanquish UHPLC system (Thermo Fisher, Waltham, MA, USA) coupled with a Q Exactive^TM^ HF-X mass spectrometer (Thermo Fisher, Waltham, MA, USA). Samples were injected into a Hypesil Gold column (Thermo Fisher, Waltham, MA, USA) at a column temperature of 40 °C, and the flow rate for gradient elution was set at 0.2 mL/min. The eluents for positive polarity mode were 0.1% formic acid and methanol, while the eluents for the negative polarity mode were 5 mM ammonium acetate (pH 9.0) and methanol. The gradient elution system is shown in Table S9.

Compound Discoverer 3.1 (Thermo Fisher, Waltham, MA, USA) was used for data processing. The metabolites with VIP > 1, *p*-value < 0.05, and fold change (FC) ≥ 1.5 or FC ≤ 0.67 were considered as differentially changed metabolites. The KEGG database (https://www.kegg.jp/kegg/) (accessed on 28 June 2023) was used for KEGG pathway analysis.

### 4.8. TMT-Based Quantitative Proteomics Analysis

TMT-based quantitative proteomics was conducted by the Novogene company (Beijing, China). In brief, protein was extracted from fine tobacco powder using precooled acetone at −20 °C for 2 h. The protein samples were then given TMT labels as follows: Mosaic_G (133N, 134N, 135N) and Mosaic_R (128N, 129N, 130N), and separated using the L-3000 HPLC system (RIGOL, Suzhou, China) with a BEH C18 column (Waters, USA). The collected fractions were subjected to UPLC-MS/MS analysis by an EASY-nLC^TM^ 1200 UHPLC system (Thermo Fisher, Waltham, MA, USA), coupled with an Q Exactive^TM^ HF-X (Thermo Fisher, Waltham, MA, USA). The precolumn and analytical column were both homemade (Novogene, Beijing, China). And the flow rate for gradient elution was set at 600 mL/min. The gradient elution system is shown in Table S10.

Proteome Discoverer 2.5 was used to analyze the raw data. The differentially accumulated proteins were defined by *p*-value < 0.05 and FC > 1.2 or FC < 0.83. KEGG pathway analysis was based on the KEGG database (https://www.kegg.jp/kegg/) (accessed on 4 September 2023), and GO enrichment analysis was based on the GO database (http://www.geneontology.org) (accessed on 4 September 2023). The proteomic data have been uploaded to iProX (http://www.iprox.org) (accessed on 5 March 2023) with the accession number PXD050349.

### 4.9. Western Blotting Analysis

For Western blotting analysis, total protein was extracted in boiling 2 × Laemmli buffer (100 mM Tris-Cl, pH 6.8, 200 mM DTT, 4% SDS, 20% glycerol) from the tobacco leaf tissue that was ground into fine powder with liquid nitrogen. The extracts were centrifuged, and the supernatant protein samples were subjected to 12% SDS-PAGE gel. Separated proteins were then transferred to the PVDF membrane. Subsequently, the PVDF membrane was incubated with monoclonal mouse anti-Flag (Sigma-Aldrich, Darmstadt, Germany) primary antibody and HRP-labeled goat anti-mouse IgG secondary antibody (Affinity Biosciences, Changzhou, China). The protein signal was imaged using a Tanon 5200 Automated Chemiluminescent Image System (Tanon, Shanghai, China) via Pierce™ ECL western blotting substrate (Thermo Fisher Scientific, Waltham, MA, USA).

### 4.10. Total RNA Extraction and qRT-PCR Analysis

Total RNA was extracted using TRIzol^®^ Reagent (Invitrogen, Carlsbad, CA, USA) and reverse-transcribed into cDNA using the PrimeScript^TM^ RT reagent Kit (Takara, Tokyo, Japan) according to the manufacturer’s instructions. qRT-PCR was performed on a QuantStudio 5 real-time fluorescence quantitative PCR system (Thermo Fisher Scientific, Waltham, MA, USA) via ChamQ Universal SYBR qPCR premix (Vazyme, Nanjing, China). *NtActin* was amplified as reference gene. The expression of target genes in the Mosaic_R region was set as “1”. The primers used for qRT-PCR are listed in Table S11.

### 4.11. Determination of Enzyme Activity

The activity of chlorophyllase, PPH, and PAO was determined by the ELISA Kits (MEIMIAN, Yancheng, China), following the manufacturer’s instructions. The absorbance was collected using a BioTek Synergy LX Multimode Reader (Agilent, Santa Clara, CA, USA).

### 4.12. Unique Molecular Identifier (UMI) Small RNA Sequencing Analysis

UMI small RNA sequencing was conducted by the Novogene company (Beijing, China). Briefly, an amount of 200 ng total RNA per sample was used as input material for the small RNA library. Sequencing libraries were generated using QIAseq miRNA Library Kit (QIAGEN, Düsseldorf, Germany) and sequenced by an Illumina Novaseq platform (Novogene, Beijing, China).

Raw data of every sample were first processed through custom Perl and Python scripts. Clean data (clean reads) were then obtained from raw data through UMI technology, and certain length of clean reads (18–30 nt miRNA) were chosen for conducting the subsequent analysis. Differentially expressed miRNAs were then screened based on *p*-value < 0.05. Target genes of DERs were predicted by TargetFinder-master. The KEGG pathway analysis of the target genes was performed on the KEGG database (https://www.kegg.jp/kegg/) (accessed on 4 September 2023). The UMI small RNA sequencing data are available in the Sequence Read Archive (https://www.ncbi.nlm.nih.gov/sra) (accessed on 22 January 2025) with the accession number PRJNA1214199.

### 4.13. Statistical Analysis

Statistical analyses of the quantitative data were performed using Microsoft Excel and IBM SPSS Statistics (version 25). Statistical significance was assessed using one-way analysis of variance (ANOVA) followed by Student’s *t*-test. Differences in statistical significance are indicated by asterisks (* *p* < 0.05, ** *p* < 0.01), and each datum was derived from three biological replicates.

## Data Availability

Data are contained within the article and Supplementary Materials.

## Supplementary Materials

The following supporting information can be downloaded at https://www.mdpi.com/article/10.3390/plants14060965/s1: Figure S1: Phenotypic changes in ASTA-mosaic tobacco plant; Figure S2: Chlorophyllase, PPH, and PAO activity in Mosaic_R and Mosaic_G regions; Table S1: List of identified metabolites in Mosaic_R and Mosaic_G regions by untargeted metabolomics analysis; Table S2: List of DCMs between Mosaic_R and Mosaic_G regions; Table S3: List of identified proteins in Mosaic_R and Mosaic_G regions by TMT-based quantitative proteomics analysis; Table S4: List of DAPs between Mosaic_R and Mosaic_G regions; Table S5: Differentially expressed proteins related to photosynthesis and carotenoid between Mosaic_G and Mosaic_R regions; Table S6: List of target genes of DERs between Mosaic_G and Mosaic_R regions; Table S7: List of KEGG pathways of target genes of DERs between Mosaic_R and Mosaic_G regions; Table S8: Gradient elution solvents for analyzing astaxanthin by UPLC; Table S9: Gradient elution solvents for positive and negative polarity mode in untargeted metabolomics; Table S10: Gradient elution solvents for TMT-based quantitative proteomics; Table S11: Primer sequences for qRT-PCR analysis.
