# Supplementary material for: Multi-Omics Analysis Provides Insights into a Mosaic-Leaf Phenotype of Astaxanthin-Producing Tobacco
Source: Plants (Basel). 2025 Mar 19;14(6):965. doi: 10.3390/plants14060965 (PMC11945019; doi:10.3390/plants14060965)
Supplement: Supplementary file 1 [file plants-14-00965-s001.zip › plants-3518924-supplementary/Supplementary Figures and Tables.pdf]

Supplementary Materials

Multi-omics analysis provides insights into a mosaic-leaf phenotype of astaxanthin-producing tobacco

Jialin Wang[1], Zaifeng Du[1], Xiaoyang, Lin[1], Peng Li[2], Shihao Sun[2], Changqing Yang[1], Yong Chen[2], Zhongfeng Zhang[1], Xue Yin[1,*], Ning Fang[1,*]

[1]Key Laboratory of Synthetic Biology of Ministry of Agriculture and Rural Affairs, Tobacco Research Institute, Chinese Academy of Agricultural Sciences, Qingdao 266101, China;

[2]Beijing Life Science Academy (BLSA), Beijing 102209, China;

[*]To whom correspondence should be addressed: E-mail: 498697153@qq.com or yinxueysh@126.com.

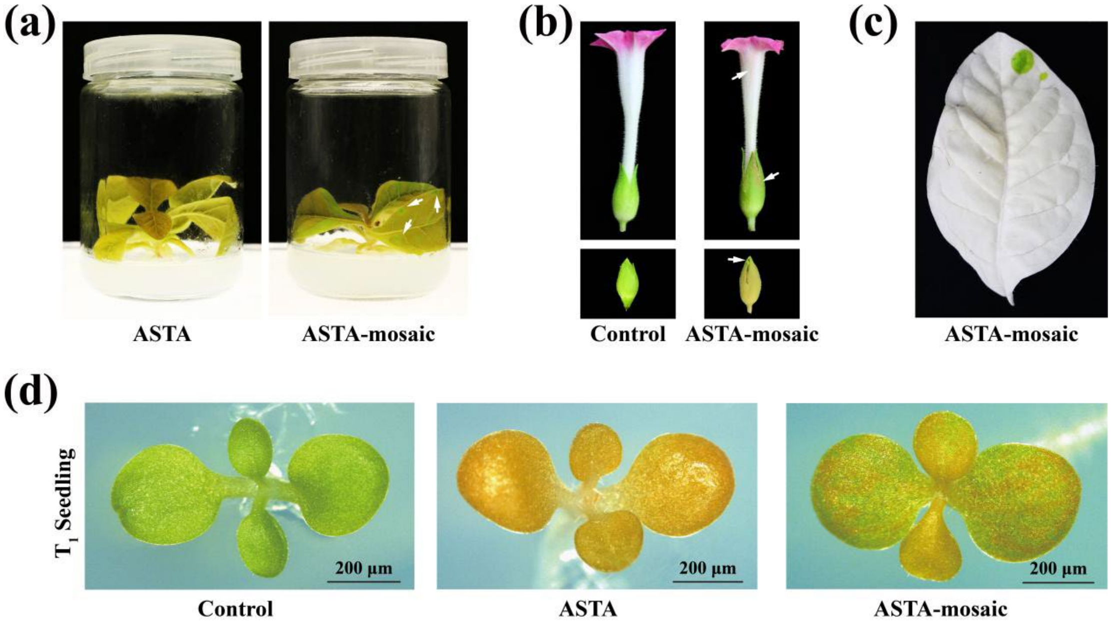

Figure S1. Phenotypic changes in ASTA-mosaic tobacco plant. (a) The phenotypic differences between red astaxanthin-producing and ASTA-mosaic plant during tissue culture. White arrows indicate green regions in leaf of ASTA-mosaic plant. (b) The change in flower and seed capsule of ASTA-mosaic plant. White arrows indicate Mosaic_R or Mosaic_G regions on the petal, calyx, and seed capsule. (c) Green regions in senescence leaf of ASTA-mosaic plant. (d) Phenotype of T1 seedlings of control, red astaxanthin-producing and ASTA-mosaic plant. ASTA, red astaxanthin-producing plant.

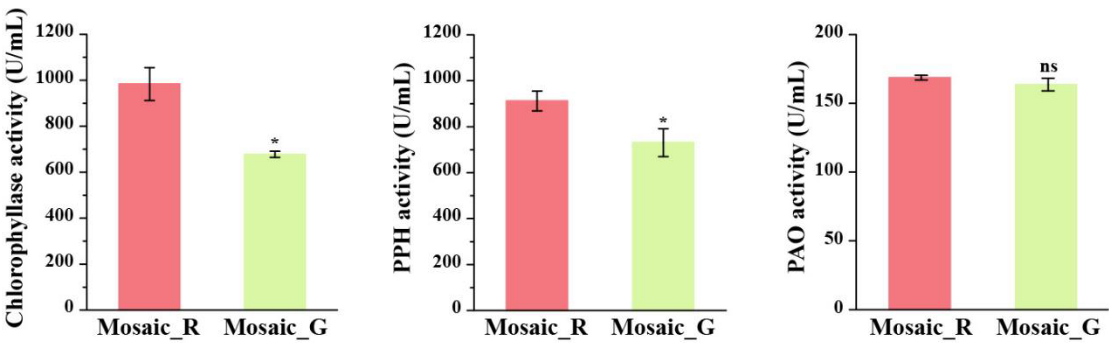

Figure S2. Chlorophyllase, PPH, and PAO activity in Mosaic_R and Mosaic_G regions (ns, $p > 0.05$; *, $p < 0.05$).

Table S1. Gradient elution solvents for analyzing astaxanthin by UPLC.

Time (min)	Mobile phase composition (%)	
	Acetonitrile	Water
0-2	40-60	60-40
2-4	60-80	40-20
4-6	80-100	20-0
6-8	100-100	0-0
8-10	100-80	0-20
10-12	80-40	20-60
12-14	40-20	60-80

Table S2. Gradient elution solvents for positive and negative polarity mode in untargeted metabolomics.

Time (min)	Mobile phase composition (%)	
	0.1% Formic acid/ 5 mM ammonium acetate (pH = 9.0)	Methanol
0	98	2
1.5	98	2
3	15	85
10	0	100
10.1	98	2
11	98	2
12	98	2

Table S3. Gradient elution solvents for TMT-based quantitative proteomics.

Time (min)	Mobile phase composition (%)	
	0.1% formic acid	80% acetonitrile with 0.1% formic acid
0	94	6
2	85	15
78.5	60	40
80.5	50	50
81.5	45	55
90	0	100

Table S4. Primer sequences for qRT-PCR analysis.

Enzyme	GeneBank ID	Primer Name	Sequence(5′- 3′)
PBGD (XP_016481887.1)	XM_016626401.1	PBGD-F	CGCAAGGGCTTCCATTGTTG
		PBGD-R	GCAAGGGCTAGGGGACTTC
MgCH (XP_016466093.1)	XM_016610607.1	MgCH-F	GAAGCGTGGAACAGCGAAAA
		MgCH-R	TAGCCCGTCTTCCCACTCAT
POR (XP_016471911.1)	XM_016616425.1	POR-F	GTTGGTTCAATTACAGGAAACACG
		POR-R	CGCTTTTGCACCGTCGAAT
Chlorophyllase (XP_016442096.1)	XM_016586610.1	Chlorophyllase-F	CGTCGCTCCTCAGTTTTCTC
		Chlorophyllase-R	CAGGACGGAGTCTAGGTTGT
PAO (XP_016498583.1)	NM_001325995.1	PAO-F	GGGTGATCAGAAGTGGGTGA
		PAO-R	GCCATCTTGGAAAAACCTGCC
PPH (XP_016437215.1)	XM_016581729.1	PPH-F	TCTGTCCTGAAGAGCGTCTA
		PPH-R	CCTCTCCTGCATTTGGGTCA
CBFD (AAV85452)	AY644757.1	CBFD-F	GGGAATTACCTTCGCAGCTTT
		CBFD-R	GATGTACCAAAGGGAATCGTG
HBFD (ABK41044)	DQ902555.1	HBFD-F	GGCTTGTGATTGGAGGAAGGA
		HBFD-R	CTCATGCTATCCACGTTCACC
